# Supplementary material for: Splice-Junction-Based Mapping of Alternative Isoforms in the Human Proteome
Source: Cell Rep. Author manuscript; Available in PMC 2020 Jan 15. (PMC6961840; doi:10.1016/j.celrep.2019.11.026)
Supplement: 3 [file NIHMS1546469-supplement-3.zip › DF2/PXD0010154/Lung-322-O76041-RPGIIVAPVLPGAYQQSHSQGYG.pdf]

A

Predicted sequence disorder and sequence features of O76041

Peptide: RPGIIVAPVLPGAYQQSHSQGYG Junction: sp|O76041|NEBL_HUMAN|ENSG00000078114|SE2|11628|chr10|20787308|20809898|−0|r85|T1 TrNovel: FALSE

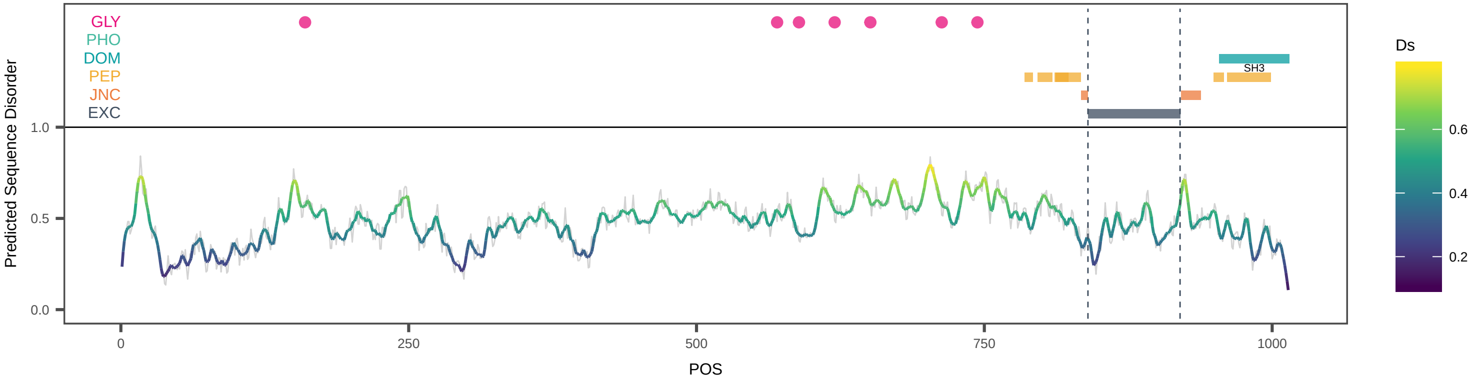

B

Distribution of sequence disorder in excised
vs. mapped and non−excised regions of protein

M−W P−value vs. mapped: 0.122 vs. non−excised: 0.000252

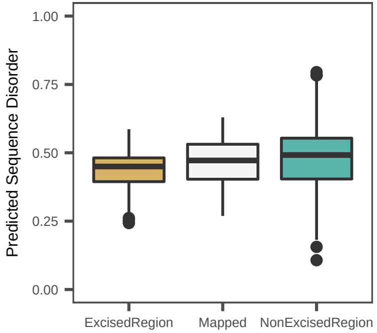

C
